# Toxic and Hallucinogenic Plants of Southern Chile of Forensic Interest: A Review

**DOI:** 10.3390/plants14142196

**Published:** 2025-07-16

**Authors:** Ramiro Díaz, Mauricio Yáñez-Sánchez, Francisco de la Fuente, Andrea Ortega, Alejandra Figueroa-Carvajal, David Gangitano, Oscar Scholz-Wagenknecht

**Affiliations:** 1 Department of Biological and Chemical Sciences, Faculty of Natural Resources, Catholic University of Temuco, Temuco 4813302, Chile; mauricio.yanez@uct.cl (M.Y.-S.); oscarscholz@gmail.com (O.S.-W.); 2 Department of Diagnostic Processes and Evaluation, Faculty of Health Sciences, Catholic University of Temuco, Temuco 4813302, Chile; fdelaf@ucn.cl (F.d.l.F.); aortega@uct.cl (A.O.); 3 Forensic Laboratory, Araucanía Region, Chilean Investigative Police, Temuco 4813302, Chile; alejandrafigueroacarvajal@gmail.com; 4 School of Medicine, Catholic University of Murcia, 30107 Murcia, Spain; dgangitano@ucam.edu

**Keywords:** Chilean poisonous plants, forensic toxicology, atropine, scopolamine, coniine

## Abstract

Several plants produce toxic and hallucinogenic metabolites, posing risks when misused due to a lack of botanical knowledge. Improper or accidental use of these plants poses a public health risk and has been associated with forensic cases involving poisoning, suicide, or drug-facilitated crimes. This review identified eight species of forensic interest that grow in southern Chile and analyzed their active compounds, mechanisms of toxicity, and documented clinical and legal cases. These selected species included both native and introduced taxa, whose main toxic agents are tropane alkaloids (atropine, scopolamine), piperidine (coniine), taxane pseudoalkaloids, and natural opiates (morphine, codeine). Most reported cases involved unintentional poisoning, mainly in children, highlighting the lack of regulation and awareness. This review revealed the need for improved forensic and clinical documentation of plant-based intoxications in Chile and greater public education regarding the toxicological risks posed by these botanical species.

## 1. Introduction

Plants synthesize toxic compounds as a defense against herbivores, pathogens, and competitors. The main hypotheses that explain this production include the optimal defense theory, which proposes a strategic allocation of defenses based on tissue value and risk; the appearance theory, which links plant visibility and longevity to the type of chemical defense deployed; and the co-evolutionary hypothesis, which posits that plants and herbivores have evolved in an “arms race,” with each developing adaptations to overcome the other’s defenses, resulting in a continuous and reciprocal interaction [1,2,3]. Recent studies also highlight synergy among secondary metabolites and their multifunctional roles in defense and signaling [4]. In fact, these chemical compounds have led to the use of plants as flavor enhancers, fragrances, insecticides, dyes, nutritional supplements, pharmacological agents, poisons, and hallucinogenic substances [5,6], as well as for inducing altered states of consciousness in shamanic rituals [7]. Certain characteristics of these plants have led to including them in important ritual and spiritual traditions, and to abuse of them on a large scale [8]. Access to plants with medical properties or to those that cause psychotropic effects is not restricted or regulated, as they are acquired through informal trade or by direct collection from nature. Plants with psychotropic actions could be used for criminal purposes such as robbery and rape. This is why the identification of these particular plants is so important [9,10,11]. Indeed, a deep knowledge of the morphology and toxicology of these psychotropic plants may help to assist in the investigation of certain criminal cases [12,13].

Some plants that are considered harmless can pose a health risk in high doses [14,15]. Although the user’s intention is not to cause harm, a lack of information about the appropriate dose, and misidentification of poisonous plants, may lead to fatal consequences, since the main organs are the target of these poisons including the central nervous system.

Identification of plant samples or fragments has been very helpful in the study of many forensic cases related to poisoning or physical harm. It may also play an important role in criminal investigations, for example, to link a person with the crime scene. The presence of plant fragments (leaves, flowers, fruits, seeds, pollen, spores, phytoliths, or roots) in clothing, stomach contents, or hair, can lead to the precise identification of plant species involved in a case [16]. This area of criminology is known as forensic botany, which provides critical evidence in criminal investigations through the analysis of plant material, pollen, and DNA. It is particularly valuable when other types of evidence are insufficient to clarify the cause and dynamics of crimes such as homicides or timber theft. Kundchhal and Inderdeep [17] present an analysis of the various domains in which forensic botany can contribute to crime resolution. Their analysis highlights the relevance of plant morphology and anatomy, palynology, dendrology, ecology, and forensic limnology.

Time is critical in emergencies caused by plant poisoning, which may require the intervention of a specialist who can provide an accurate diagnosis and appropriate treatment. In this context, knowledge of specific plants that grow in a particular region is very important for forensic science, and for medicine in general. There are many wild and ornamental plants that may have toxic effects on humans [18]. Knowing how to recognize these plants, their toxic properties, and their clinical symptoms provides great help in medical assistance for a timely application of treatment. In the case of forensic science, understanding toxic effects may provide evidence for crime investigations [19].

Poisoning by toxic plants can be classified based on the type of toxin (active component), the affected organ system, and the plant species involved. Another classification includes accidental ingestion, voluntary ingestion, substance abuse-related poisoning, and deliberate suicide [20,21]. The level of toxicity depends on the species used, route of administration, plant age, and the part of the plant consumed. Additional critical factors include the ingested dose and the individual’s susceptibility [22].

Voluntary ingestion is frequently reported in suicide cases. For instance, two adult men (44 and 56 years old), both with a history of substance abuse, were found dead in their homes. In both cases, there was evidence of *Aconitum* plant ingestion, with plant remnants and cups containing a brownish liquid consistent with an herbal infusion found nearby [23]. In contrast, involuntary ingestion is more common in children or results from plant and fungal misidentification in adults [24]. In forensic science, the term “chemical submission” refers to the use of psychoactive substances for criminal purposes, intended to manipulate a victim’s will and alter their behavior. These substances are typically colorless, odorless, tasteless, fast-acting, and have a short half-life, producing anterograde amnesia. They are often administered in beverages to mask their sensory characteristics [25,26,27]. Such practices are frequently associated with sexual offenses [11].

In Chile, few cases involving toxic plants have been documented in the scientific literature. One of the rare reports describes the intoxication of a 23-day-old infant due to an infusion made from the fruit of *Illicium verum*, reported in 2003 [28]. Conversely, monographs, statistical reports, scientific publications, and newspaper articles have identified various plants containing toxic compounds, providing their botanical and chemical characterization, geographic distribution, and adverse effects on humans and animals [29]. However, rigorously documented forensic cases remain scarce [2]. Voluntary and involuntary poisonings that may be relevant to forensic investigation frequently appear in the media, particularly when associated with unlawful acts. For example, in a recent involuntary poisoning case, Chilean newspapers reported on 21 May 2025, the death of a Chinese national after ingesting leaves of *Nicotiana glauca* in a salad, a wild plant native to north-central Chile. Due to the recency of the incident, no official forensic report is yet available.

In ancestral cultural contexts, psychoactive plants are primarily consumed by self-taught individuals who have learned from traditional Mapuche-Huilliche ethnomedicine yet often lack basic pharmacological knowledge. These plants are commonly used by machis (shamans) [30]. A machi is the person who officiates religious ceremonies in Mapuche-Huilliche communities. A machi is a spiritual leader in Mapuche-Huilliche communities, whose ancestral territories are located in the present-day Araucanía and Los Lagos regions of southern Chile. Machis frequently inhale smoke from canelo leaves (*Drimys winteri*) or ingest preparations containing *Datura stramonium* to induce trance and ecstatic states. The use of *Latua pubiflora* is also frequent, as it is traditionally associated with kalkus (sorcerers) and malevolent rituals. The fruit of *L. pubiflora* is extremely toxic and capable of inducing delirium and cardiorespiratory failure even at low doses. Due to this association with harmful practices, machis have served as custodians of this proto-botanical knowledge, transmitting it across generations. However, such practices have declined over time due to a broader disconnection from nature. These are only a few examples of plants used in Mapuche ethnomedicine [31].

In southern Chile (36°00′–51°00′ S and 67°00′–71°00′ W, bordering the Pacific Ocean), at least 26 plant species, widely distributed in both natural and urban areas contain toxic substances with poisonous and/or hallucinogenic effects. These plants may be used alone or in combination with other substances for criminal purposes.

Several toxic plants, including *Datura stramonium*, *Latua pubiflora*, *Atropa belladona*, *Brugmansia vulcanicola*, *Conium maculatum*, *Colchicinum autumnale*, *Taxus baccata,* and *Papaver somniferum*, have been reported in the literature in association with forensic cases, either as whole plants or through their secondary metabolites [32]. Many of these plants are traditionally used in folk and ethnomedicine. When administered in low doses, some are used for pain relief; however, they often contain neurotoxins that affect the central nervous system, impairing motor and sensory coordination, and causing symptoms such as delirium, unconsciousness, and coma. Others contain cytotoxins and metabolic toxins that target specific organs, including kidneys, liver, heart, and lungs, leading to a spectrum of effects from mild symptoms to organ failure.

The aim of this review is to describe selected forensic cases and to analyze the toxicological characteristics, geographic distribution, and biological effects of eight toxic plant species found in southern Chile, emphasizing their relevance to public health and safety. Additionally, this review seeks to highlight the need for systematic and scientific reporting of poisoning cases in Chile, in order to support law enforcement and medical personnel in effectively responding to intoxication events.

## 2. Methodology

### 2.1. Information Search and Selection Strategy

This review was conducted following PRISMA guidelines, adapted for non-systematic reviews. A comprehensive search of scientific literature was carried out in the databases PubMed, Scopus, Web of Science, ScienceDirect, and Scielo, covering publications up to April 2025.

Peer-reviewed articles, scientific books, and institutional reports published in English or Spanish were considered. The selection focused on the botany, toxicity, and forensic relevance of plant species found in southern Chile. Boolean operators (AND/OR) were used to combine search terms as listed in Table 1. No restrictions were applied regarding publication year.

All records were manually screened by title and abstract. Duplicates and non-relevant articles were excluded.

### 2.2. Selection of Plants and Inclusion Criteria

The selection of plant species was based on the following inclusion criteria:

- Occurrence in southern Chile (36°00′–51°00′ S).
- Presence of documented toxic properties and known bioactive compounds.
- Forensic relevance, including the following: Voluntary or involuntary poisoning, Suicide cases, Chemical submission or criminal use.
- Availability of scientific information on at least one of the following: toxic compounds, clinical effects, forensic reports, or ethnomedicinal usage.

Studies unrelated to forensic toxicology or lacking scientific evidence were excluded.

### 2.3. Analysis and Synthesis of Information

The selected articles were reviewed and analyzed collaboratively by all authors. For each plant species, the following information was synthesized:

- Botanical information: scientific and common names, morphological traits, habitat.
- Toxicological profile: toxic compounds, mechanisms of action, dose-response data.
- Clinical manifestations: symptoms, affected systems, time of onset.
- Forensic cases: description of real cases, intoxication context, outcomes.
- Ethnobotanical relevance (when applicable).

To clarify the use of key terms in this review, Table 2 summarizes the main toxicological and pharmacological categories applied to the selected species.

This structured summary enabled a qualitative analysis of the selected species with emphasis on their impact on public health and forensic investigation in southern Chile.

## 3. Botanical Species Involved in Cases of Forensic Interest

**Figure 1:**
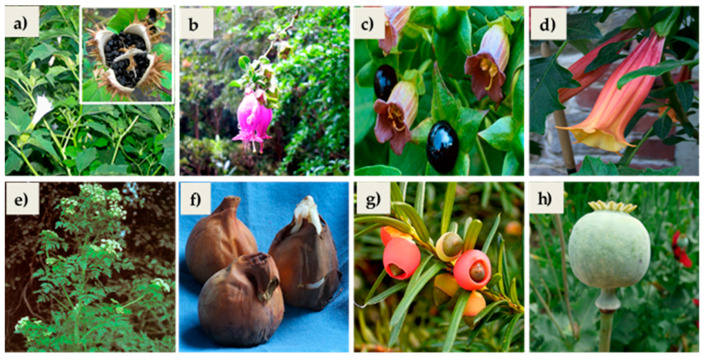
(**a**) Leaves, flower, and unripe fruit of *D. stramonium*; (**b**) Leaves and flower of *L. pubiflora*; (**c**) Flower and fruits of *A. belladonna*; (**d**) Flowers and leaves of *B. vulcanicola*; (**e**) Whole plant of *C. maculatum*; (**f**) Seeds and bulbs (corms) of *C. autumnale*; (**g**) Leaves and fruit of *T. baccata*; (**h**) Fruit of *P. somniferum*.

Table 3 lists the plant species, including their botanical data and the most toxic parts of each. Table 4 presents the toxicological aspects and the pharmacological properties of the plants. Figure 1 shows images of the toxic parts of each plant species.

The severity classification criteria used were as follows [18]:

High: A moderate dose may cause severe symptoms (e.g., seizures, coma). Requires urgent medical attention. Long-term effects are possible but uncommon.

Very high (lethal): A low dose may cause death. Human lethality has been documented. No specific antidote or effective treatment is available.

Each selected species is presented below following a standardized structure that includes (i) a brief botanical description, (ii) distribution in Chile, (iii) main toxic metabolites, (iv) mechanism of action, (v) clinical or forensic cases, and (vi) forensic relevance. This unified framework aims to facilitate comparative analysis and improve the clarity and consistency of toxicological and forensic information presented.

### 3.1. Datura Stramonium

*Datura L*. is a genus of the Solanaceae family. Plants of this genus are annual herbs, small or large shrubs, or small trees distributed mainly in tropical and subtropical regions. They have been used in traditional medicine to treat respiratory diseases, stomach pains, and rheumatism. These plants are also known for the toxicity of some of their chemical compounds [33,34,35,36].

*Datura stramonium*, known throughout South America as chamico or miyaya, is an annual herbaceous plant growing up to around 1.2 m high. This plant is native to the whole continent, and easily distinguished by its spiny seed capsule. In Chile, it grows from the O’Higgins to the Los Lagos regions, in inland valleys at altitudes up to around 700 m above sea level (masl). It releases a strong odor and has white to violet-blue trumpet-shaped flowers. It is used as a hallucinogenic drug as it contains various anticholinergic agents [33,35].

**Figure 2:**
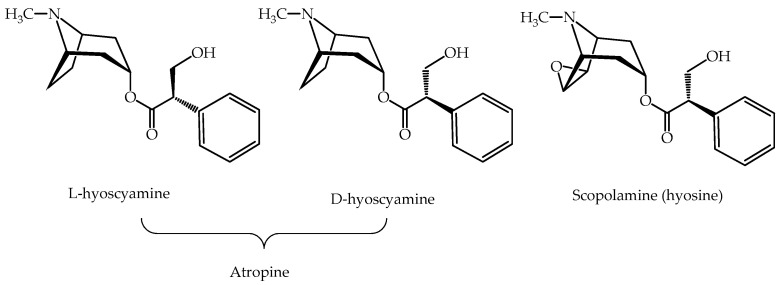
Main psychoactive chemicals present in species of Solanaceae family.

The chemical structures of the two main toxic components, namely, the tropane alkaloids atropine, which is a racemic mixture of D-hyoscyamine and L-hyoscyamine, in which the levo isomer (L) is the biologically active form [37], and scopolamine (also called hyoscine) [38,39], are shown in Figure 2. Both are physiologically active in doses between 0.3 and 1 mg in adults. Consumption of any part of the plant causes poisoning and consequently anticholinergic syndrome [40,41,42], and prominent antimuscarinic effects. The antimuscarinic effects result in altered states of consciousness, a wide range of visual and auditory hallucinations, and seizures. This may lead to deep coma and even death from general decompensation since atropine causes vasodilation and tachycardia [43]. The peripheral muscarinic receptors are associated with innervation by the parasympathetic nervous system, and their blockage causes dry mouth, constipation, and dilated pupils, leading to blurred vision [8,44]. This type of poisoning may occasionally be difficult to diagnose [45]. Scopolamine may induce malfunctioning of the memory circuit in the central nervous system and dysregulation of the cholinergic neuronal pathway [41].

Children are highly susceptible to these toxic effects, which even in very low doses produce severe symptoms in the central nervous system. Scopolamine can produce severe respiratory failure and cardiovascular collapse, as well as fever, nausea, blurred vision, and hallucinations [43]. This is mainly due to children’s lower body weight and their less effective liver detoxification in comparison with adults [46]. Unfortunately, diagnosis tends to be delayed unless information or proof of ingestion is received early. The toxic effect starts after 30 min and can last for up to 48 h. Although scopolamine poisoning does not usually lead to death, it represents 0.1% of all deaths caused by poison in the US and up to 35% of deaths caused by plant consumption [47].

In Nigeria, a case was reported of a 14-year-old boy who was admitted to hospital with symptoms of verbal incoherence, restlessness, optical hallucinations, inability to recognize family members, and irrational behavior. On examination, the subject presented confusion, fever, dry mouth, dilated pupils, tachycardia, and systolic hypertension. Diagnosis of *D. stramonium* poisoning was based on clinical symptomatology and mainly on the testimony of an eyewitness who saw the boy ingesting the plant material [48]. In similar cases, a 20-month-old boy and two others, ages five and three years old, were admitted to an emergency room with symptoms of poisoning. The first presented agitation and restlessness, although examinations (neurological, hemodynamic, biological, and cardiological) gave normal results. The second child presented a state of agitation, confusion, bilateral mydriasis, and hyperthermia (39 °C); other examinations (hemogram, hemostasis assessment, blood ionogram, liver and kidney assessment), produced normal results. The third one also presented with generalized rigidity followed by rhythmic flexion and extension of the extremities, with loss of consciousness lasting 2 to 3 min. The testimony of family members was critical in determining that both children had ingested an indeterminate quantity of *D. stramonium* leaves and seeds [49,50]. In another case, a whole family was hospitalized with similar symptoms after eating dolma, a traditional Turkish dish, which had been prepared with *D. stramonium* flowers. All family members had anticholinergic symptoms and were treated for anticholinergic poisoning based on the origin of the ingredient used [51]. Not all cases of poisoning present the same symptoms. For example, in the case of two brothers aged five and four years old, who drank tea prepared with *D. stramonium* leaves and flowers, the first boy presented agitation, hallucinations, and mydriasis, and improved rapidly under treatment with benzodiazepine, while the second brother presented a state of coma, mydriasis, and Babinski signs. A stomach pump was applied and charcoal was administered. He was intubated and placed on mechanical ventilation until he recovered consciousness [52].

The broad distribution of the plant, its strong toxicity, and the possibility of being ingested casually lead to frequent cases like the above-mentioned, especially related to the ingestion of seeds [53]. Seeds are the most toxic part of the plant, followed by the roots, stems, leaves, and flowers [54]. Ingestion of just ten seeds is sufficient to cause psychoactive effects, and 125 to cause heart failure [45,53].

Scopolamine, also known as “burundanga” among those who abuse the substance, is a tasteless, odorless alkaloid that is easily absorbed in the digestive tract; it can be administered by oral or dermal route, or by inhalation [55]. Antidepressant effects have been reported in studies of scopolamine. In one study, which included 62 subjects aged between 18 and 55 years old with depression or bipolar disorders, the subjects were separated into two groups, one presenting resistance to the drug and a control group who had never been exposed to psychotropic agents. All were given, at random, either scopolamine or a placebo. In both groups, it was found that scopolamine caused substantial effects in reducing depressive symptoms [56]. One of the most common effects of scopolamine is loss of memory and a feeling of willpower [57,58,59,60,61]. Although scopolamine does not cause as many deficiencies as those observed in Alzheimer’s patients, it produces alterations in memory similar to those observed in patients of advanced age [59,62]. In a recognition memory test, two groups of monkeys (test and control groups) were subjected to the effects of the scopolamine; both groups showed disabilities in executing tasks, proportional to the doses of the drug administered [63]. Scopolamine has been administered to diminish memory in rats, with the object of determining the effect of certain extracts and drugs on memory [64,65,66,67].

In the forensic context, cases have been reported of outbreaks of anticholinergic toxidrome associated with the involuntary contamination of spinach leaves with *D. stramonium*. More than 100 people experienced symptoms consistent with poisoning with the most abundant alkaloids derived from this plant [68]. There are also reports of chemical submission, in which this drug was used to commit various crimes such as theft, sexual offenses, and other criminal actions [69]. In Spain, a case was reported of a woman who consulted a supposed shaman. She voluntarily drank a potion containing scopolamine. Once drugged, she was subjected to repeated sexual abuse by the man. After this case was made public, 38 other women reported having been raped by the same person [70]. Symptoms like blurred vision, difficulty in walking, abdominal pain, and dryness of the mouth, followed by a period of amnesia, are described after the intoxication [69].

In Great Britain, a case was documented of a mother who administered scopolamine to her children for 10 years in tablets called Feminax, an over-the-counter medicine. This product is generally used to relieve headaches, toothache, and period pains. A similar case was reported about three children who were frequently beaten by their mother, who further forced them to swallow four to ten Feminax tablets (Bayer, Berkshire, UK) [71].

### 3.2. Latua Pubiflora (Griseb)

A shrub or small tree 2 to 10 m high, belonging to the Solanaceae family, it is the sole member of its genus. The species, known as latué or palo de brujos, is endemic to Chile. It grows in wet forests at heights of 300–900 masl in Los Ríos and Los Lagos regions [72].

No scientific reports have documented criminal cases involving this plant. However, alkaloids similar to those found in *Datura stramonium* have been identified. Consequently, its effects and symptoms are expected to be comparable, including visual hallucinations, delirium, trance-like states, and seizures—due to its strong anticholinergic activity at high doses. Symptoms of poisoning may appear immediately after ingestion or be delayed for up to 24 h. The primary alkaloids identified include atropine and scopolamine. Other alkaloids found are 3α-cinnamoyloxytropane, 3α-apotropoyloxytropane, norhyosciamine, 6-hydroxy-3-O-acetyltropine, hygrine, 3-O-acetyltropine, 7-hydroxy-3-O-acetyltropine, scopine, 3α-apotropoyloxytropane and 7-hydroxyhyoscyamine [73,74,75].

### 3.3. Atropa Belladonna

Commonly called belladonna, this plant is a perennial shrub of the Solanaceae family, native to Europe, North Africa, and Asia, which grows up to 1.5 m high. It will grow almost anywhere in Chile, including the far south. Its flowers are purple with green or greenish tints, a slight odor, and a bell-like shape. It is found in gardens as an ornamental plant for its large fruit-sweet-tasting berries of approximately 1 cm in diameter, which are shiny black when ripe. Its name, Atropa, alludes to the Greek Fate named Atropos who was responsible for cutting the thread of life [76]. This plant was used to poison Livia, the wife of the emperor Augustus, and Agrippina, the wife of Claudius [77].

It has been used since ancient times for medicinal, cosmetic, aphrodisiac, and narcotic purposes. It is rich in tropane alkaloids, mainly atropine and scopolamine [54,78]. Other identified alkaloids are belladonnine, norhyoscyamine, apoatropine, and 6-β-hyoscyamine [79]. Calystegine (a potent inhibitor of β-glucosidase) has also been found in all parts of the plant, mainly the upper leaves [80]. A recent study of the root reported 13 compounds, including scopoletin, scopoline, and tropic acid, a precursor of the chemical synthesis of atropine and hyoscyamine [81].

Similar symptoms to those described for *D. stramonium*, such as anticholinergic collapse, effects on the central nervous system, and mydriasis, have also been described for the consumption of *A. belladonna* [19,82]. The consumption of just one berry can cause intoxication, ingestion of three to four berries is sufficient to endanger a child’s life, while 10 berries may be toxic for an adult. The symptoms generally appear 10 min to 2 h after ingestion [5]. Since children are more sensitive to alkaloids, a very small quantity can produce severe symptoms of poisoning, and in some cases death [83].

In 2010, a case was reported in France of a 2-year-old child who was admitted to pediatric emergency services with symptoms of atropine syndrome, including behavioral alterations, hallucinations, reddening of the face, dryness of the mouth and lips, bilateral mydriasis, balance problems, agitation, and confusion. A few hours earlier, the child had eaten wild berries from a bush. A sample from that bush was submitted to the aid center and helped to identify it as *A. belladonna*. The timely identification of the plant helped to provide the specific medical treatment that eventually saved the child’s life [84].

A retrospective review in 2016 reported some cases of children treated after ingestion of anticholinergic agents between 2001 and 2010, in the Hospital for Sick Children of Toronto. Ten children were identified, aged 15–18, who had consumed some product containing atropine. All children presented severe anticholinergic symptoms; two cases were suicide attempts [85]. In adults, the symptoms may include confusion, somnolence, dizzy spells, anxiety feelings, visual disability, inability to talk, etc. In one case reported, these symptoms began 10 min after the ingestion of 30 drops (approx. 1.5 mL) of a homeopathic drug containing an extract of *A. belladonna*. This drug was prescribed by an alternative medicine physician to treat stomach ache [86].

### 3.4. Brugmansia spp.

The genus *Brugmansia*, of the Solanaceae family, contains eight species distributed in America, Europe, Africa, and Asia. The plants of this genus are used in traditional medicine to treat inflammation, rheumatoid arthritis, wounds, skin infections, headache, asthma, colic, pain, etc. [87,88]. There are numerous cultivated varieties, divided into groups according to the shape of their flowers [89]. For this reason, some are commonly known as “angel’s trumpet”. Being members of the Solanaceae family, these species are toxicologically related to *D. stramonium*, with a high content of tropane alkaloids [89,90,91]. Angel’s trumpet, with a height of up to 4 m, is a perennial tree endemic to South America, especially the tropical and subtropical regions. In Chile, it is known as floripondio and it can be found from the extreme north to the Los Lagos region (in south-central Chile), at altitudes up to 600 masl. In southern Chile, it is cultivated for ornamental use due to its striking flowers. The most popular hybrid is *Brugmansia* × *candida*, produced by crossing *B. aurea* × *B. versicolor*. It has long, hanging, trumpet-shaped flowers, yellow or white in color, with a height of between 18 and 23 cm. The flowers are quite aromatic, especially at night. It is frequently found as an ornamental plant in gardens and parks. In addition to its use in traditional shamanic religious ceremonies among the indigenous peoples from South America, it is also consumed as a powerful hallucinogenic. An infusion with four leaves or one flower causes psychoactive effects in adults, which can last for more than 24 h, resembling *D. stramonium* and *A. belladonna*. Poisoning with alkaloids contained in species of the genus *Brugmansia* provokes mydriasis and delirium with visual and auditory hallucinations [92], and may even cause anticholinergic collapse in most severe cases [93]. Various cases of poisoning have been reported, mainly from accidental ingestion by children and recreational use by adolescents. The most frequent symptoms are alterations in orientation, affective lability, incoherent thoughts, struggling ideas, tangential thoughts, scenic or threatening visual and auditory hallucinations, agitation, and anxiety [94]. Patients tend to experience amnesia after ingestion [90,95]. A 60-year-old woman, after drinking an infusion of leaves from an unknown plant in her garden, presented acute confusion, delirium, and agitation, as well as urinary retention, mydriasis, and sinus tachycardia. The unknown plant was identified by botanical analysis of the leaves as *Brugmansia suaveolens* [96]. In another case, a 64-year-old Korean woman presented acute mental alterations caused by inadvertent ingestion of the petals of angel’s trumpet flowers, used as a garnish for a traditional Korean dish. Physical examination and blood tests revealed no abnormal conditions and she recovered her normal level of consciousness after 10 h. At that moment, she remembered ingesting the flowers, which might explain her symptoms [97]. Additionally, another symptom that has been observed was unilateral anisocoria. A 3-year-old girl was taken to an emergency service with her right pupil very dilated. According to her parents, she had had no contact with drugs and there was no evidence of trauma or lesions. However, they reported that she had experienced several similar episodes lasting one to two hours in the last year after playing in the back garden. On this occasion the symptoms lasted for several hours, with blurred vision in the affected eye. After discarding various possible causes of the symptoms, it was suggested that they might have a pharmacological cause. There are many substances with antimuscarinic activity that can cause anisocoria due to topical exposure, leading to pharmacological mydriasis; they include scopolamine, atropine, and homatropine [98]. The same effect may occur with environmental exposure to plants, including species of *Datura* and *Brugmansia*. In the previously described case, her parents were sure that there was no possibility of exposure to any exogenous antimuscarinic poison. However, parents immediately identified the angel’s trumpet as one of the plants of their garden [99].

### 3.5. Conium Maculatum

**Figure 3:**
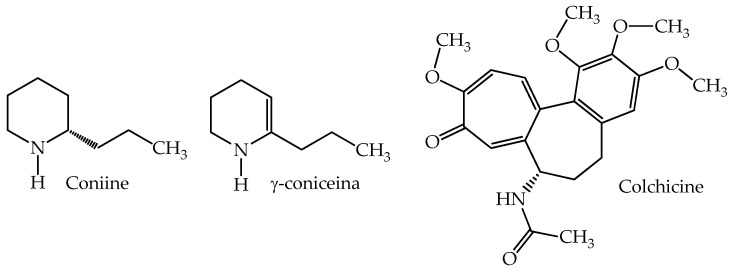
Main toxic chemicals presents in the species *C. maculatum* and *C. autumnale*.

Cicuta (hemlock) is a plant belonging to the Apiaceae family and is one of the most poisonous ones. It is the third most frequent plant involved in poisoning [100]. It is a biennial plant, of erect growth, found in Chile from the Metropolitan area to the Los Lagos regions, preferably at low elevation in cool, damp, nitrophilous environments. The stems, which can grow up to 2.5 m, are hollow and striated, with purple spots near the base and multiple branches higher up. It has small white flowers, grouped in umbels of around 10 to 15 cm diameter. It is easily identifiable by its large size, by the reddish or purple coloring of the sprouts and stems, and, especially, the fetid, urine-like odor emitted by leaves. Its medical use has been known since ancient times as an analgesic for diseases such as arthritis and neuralgia. However, its medical use was banned due to the small difference between therapeutic and toxic doses. Moreover, it was well-known as an effective method of execution. Eight piperidine alkaloid components have been identified in different plant aerial tissues (coniine, N-methylconiine, γ-coniceine, conhydrine, conhydrinone, pseudoconhydrine, N-methylpseudoconhydrine, and 2-methyl-pipiridine) [101]. Figure 3 shows the chemical structures of the most abundant components: coniine and γ-coniceine, the latter being considered the most poisonous [102,103]. A recent study demonstrated the absence of alkaloids in the roots [104].

These alkaloids act as powerful stimulants of the central nervous system. They are antagonists of gamma-aminobutyric acid (GABA), blocking its action and causing neuronal depolarization. In mammals, they stimulate the cortex, with the most pronounced effects in the mesencephalon and the medulla. These same effects have been previously described for picrotoxin and strychnine. The initial stimulation of the ending of motor nerves and CNS resulted in paralysis and central depression. This is followed by increased heart rate, excessive salivation, hyperventilation, urination, frequent defecation, and finally, coma and death by respiratory failure [105]. These symptoms are very similar to those caused by nicotine poisoning. In mice, it has been observed that coniine provoked fasciculations of the skeletal muscles and seizures, followed by paralysis and subsequent death by respiratory failure [106].

In humans, hemlock poisoning has been recorded since the time of Socrates in ancient Greece [107]. Hemlock produced accidental poisoning because its leaves were confused with plants such as fennel (*Foeniculum vulgare*), celery (*Apium graveolens*), and parsley (*Petroselinum crispum*), its roots with those of parsnip (*Pastinaca sativa*), or its seeds with those of aniseed (*Pimpinella anisum*). As a lethal poison, effective in low doses, the juice or extract was also used as a method of executing criminals and political prisoners—including Socrates himself, who was forced to drink hemlock in 399 BC.

Cases of poisoning with *C. maculatum* have been documented in different countries. In Australia, two young men in a state of drunkenness died in 1993 after consuming *C. maculatum* leaves. The autopsy revealed hepatic and pulmonary congestion in both bodies. Toxicological results confirmed the presence of 11-carboxy tetrahydrocannabinol, ethanol, and γ-coniceine in the urine of both victims and the stomach contents of one of them. In another reported case [108], a group of four children ingested *C. maculatum* leaves which they confused with an edible local plant called “carrot grass”. When they became aware of the bad taste and smell, three of them spat the leaves out, but one, aged three years old, swallowed the whole portion. Seventy min after ingestion, the child fell asleep; 90 min later, they were found dead, and all efforts at revival proved useless. In another case, a four-year-old boy and his father ingested the green leaves of a “wild carrot”. Within 30 min, the child became somnolent, his heart rate reached 100 per min, and his pupils became miotic. Two hours later, the child threw up a green liquid. After treatment with a stomach pump, activated carbon, and infusions, he recovered consciousness within 4 h [109]. In a similar case, a 6-year-old girl ingested a plant that she found in a garden, thinking it was parsley. Two hours later she was taken to an emergency service with a burning sensation in the mouth, increased salivation, trembling hands, and loss of balance while walking. After examination, the plant proved to be *C. maculatum* [110]. In Iran, three adult men aged 30, 32, and 50 years old who worked as shepherds, with a record of addiction to opioids, ate a forest plant along with vegetables for their lunch. Within a few minutes, they experienced muscular weakness, particularly in the legs, abdominal pain and gastrointestinal symptoms, lethargy, dizziness, nausea, vomiting, intense diaphoresis, and diminished consciousness. Botanical analysis of the plant proved to be *C. maculatum* [111]. In two other reported cases, a 27-year-old woman with a record of bipolar disorder and previous suicide attempts was found dead in a sleeping bag in a relatively isolated forest area of northern California; the other case was a 44-year-old man with alcohol problems, who was found dead in the kitchen of a trailer in eastern Pennsylvania. In both cases, the botanical evidence indicated the presence of *C. maculatum*, and the toxicological evidence showed the presence of coniine in blood and stomach contents at a dose that caused death [112]. In a recently reported case, a 50-year-old woman used an alcoholic extract of hemlock as self-medication. Three to four hours later, she was admitted to the emergency room. Although her health rapidly deteriorated, it was possible to save her life [113].

Symptoms of toxicity in animals are similar to those observed in humans, except for the fact that humans develop acute kidney failure. In human and animal species, hemlock poisoning inhibits fetal movements. Indeed, the accumulation of piperidine alkaloids in the blood of the fetus diminishes the sensitivity to nicotinic acetylcholine receptors. Thus, this causes serious anomalies in fetal development which can lead to various types of malformations, such as cleft palate and cleft lip [114]. In humans, this inhibition of fetal movements was associated with craniofacial defects, pulmonary hypoplasia, and other abnormalities in newborns [115].

*Conium* alkaloids produce similar effects to those of nicotine, including sialorrhea, mydriasis, and tachycardia followed by bradycardia. Coniine can produce rhabdomyolysis, either by a direct toxic effect on the skeletal muscle or by a similar action to that of strychnine on the central nervous system [116].

### 3.6. Colchicinum Autumnale

Although this plant belongs to the Liliaceae family, it does not grow in southern Chile. However, its active principle, colchicine (Figure 2), is available in commercial pharmacies. This tricyclic alkaloid can be extracted from two natural sources, *Colchicum autumnale* [117] and *Gloriosa superba* [118]. It is used in medicine to treat acute cases of gout since it has anti-inflammatory, anti-mitotic, and anti-carcinogenic activity [119,120]. It was also investigated as a possible treatment for SARS-CoV-2 [121].

Colchicine is considered a serious threat to life in cases of poisoning since it blocks cell division by inhibiting mitosis. In severe cases, the symptoms start with acute abdominal pains, severe vomiting, diarrhea, and dry tongue, leading subsequently to dizziness, metabolic acidosis, liver failure, kidney failure, lung failure, and finally, multi-systemic organ failure. These symptoms have been compared with those produced by arsenic poisoning [122]. In cases with successful outcomes, typical treatment included providing activated carbon to the victim and/or a stomach pump. Additionally, the patient received support for cardio-circulatory, lung, and kidney symptoms, if required [123]. Cases of fatal poisoning caused by cardiotoxic effects or mitosis inhibition have been reported. A child aged nearly three years old died of cardio-respiratory and hepatic failure after ingestion of *C. autumnale* (serum concentration of colchicine 7 μg/L, toxic level > 5 μg/L). In some fatal cases of accidental poisoning, patients confused wild garlic (*Allium ursinum*) with *C. autumnale*, due to the similar morphology of both plants. In all cases, symptoms included gastrointestinal problems (abdominal pain, prolonged vomiting, diarrhea), acute kidney insufficiency, coagulopathy, myocardial necrosis, and death due to respiratory difficulty and cardiopulmonary arrest [18,124,125]. A similar case was reported in Japan, in which *C. autumnale* was confused with *Allium victorialis*, an edible wild plant popular in northern Japan [126]. In France, two cases of suicide by ingestion of colchicine-containing medications derived from Colchicine Houdé^®^ (Hoechst-Houdé, Paris, France) have been documented. One case involved a 39-year-old woman who ingested 40 tablets of 1 mg each, and the other, a 45-year-old man who drank a bottle of oral suspension of Colchicine Houdé^®^ and one of Colchimax^®^ (Hoechst-Houdé, Paris, France). [127]. In southern India, where *C. autumnale* grows naturally, the suicide of a 24-year-old man by ingestion of parts of this plant was reported. Two cases of murder using this plant have also been reported in India [128].

### 3.7. Taxus Baccata

**Figure 4:**
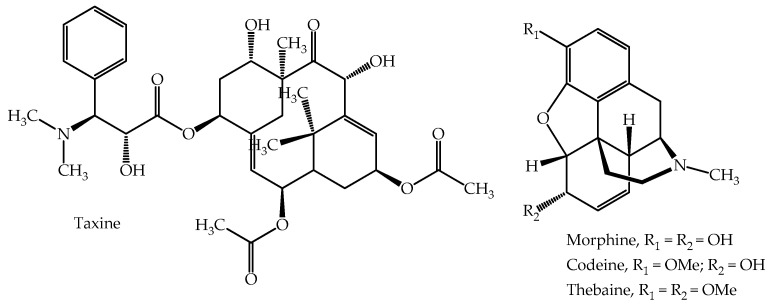
Main toxic components or chemicals present in the species *Taxus baccata* and *Papaver somniferum*.

This tree belonging to the Taxaceae family is native to Europe. In Chile it is known as Tejo; its common English name is yew. It is a small to medium evergreen tree which can live many years and it may grow as high as 20 m. It has narrow, dark-green lanceolate leaves measuring one to four cm in length and two to three mm in width, and bears a delicate, bell-shaped, bright red “fruit”, which is, in fact, an arillus that forms around the seed. It is, therefore, a popular ornamental tree and has been widely planted in parks and gardens in many countries, since it is highly adaptable to different types of climate at elevations up to 800 masl [129]. Except for the fruit, the rest of the tree tissues are highly poisonous, containing the taxane pseudo-alkaloid taxine [130]. The chemical structure of this alkaloid is shown in Figure 4. Symptoms described for overdose of taxine include nausea, dizziness, abdominal pain, shallow breathing and tachycardia, and even coma and seizures in acute cases [82]. Another toxic component present in the leaves is 3,5-DMP (3,5-dimethoxyphenol), which can be used as a marker to identify poisoning with *T. baccata* leaves [131]. Death occurs as a result of respiratory paralysis with cardiac arrest in diastole. Just 1 g of leaves contains around 5 mg of taxine; clinical signs appear around two or three h after ingestion [5]. The lethal dose is 0.6 to 1.3 g of leaf per kg bodyweight, equivalent to 3.0 to 6.5 mg of taxine/kg for an adult weighing 70 kg. The young stems contain the taxoids 10-deacetyltaxezopidine G, taxezopidine G, lariciresinol and taxiresinol [132]. Toxicity due to ingestion of *T. baccata* has been documented in ruminants [133,134], where a dose of 5 g of taxine per kg live weight causes cardiac failure and sudden death [135].

Previous investigations reported 22 cases of fatal poisoning involving *T. baccata*. The mean age of the victims was 29.6 years, and 45% were male. In fatal poisoning cases, 55% were dead when found. The time from ingestion to death was between two and 24 h. Fifteen cases were suicides, and one death was due to accidental ingestion. It could not be established whether the other six cases were accidental or suicides [136].

In general, the most frequent symptoms observed by emergency services are gastrointestinal and neurological disorders and subjective cardiovascular symptoms. The most frequent clinical findings were tachycardia and hypotension [137].

Cases of suicide and attempted suicide with *T. baccata* had in common the presence in the blood of 3,5-DMP, taxine A, 2-deacetyltaxine, monoacetyltaxine, taxine B (isotaxine B), etc. [138,139], as well as states of unconsciousness, bradycardia, tachypnea, and cyanosis [140] or sudden death after an episode of respiratory insufficiency and cardiac arrest [141,142,143]. In all cases, botanical investigation identified *T. baccata* remains in the victim’s surroundings and/or in gastric samples.

In cases of fatal poisoning where the victim was found dead, other causes of death were often suspected, such as epileptic attack or cardiac arrest, but the autopsy revealed plant material in the stomach and intestines. Botanical analysis was critical for the identification of *T. baccata* in these cases [144,145]. Toxicological or forensic analysis was carried out by verifying the presence of 3,5-DMP [146].

### 3.8. Papaver Somniferum

*Papaver somniferum* is an herbaceous plant of the Papaveraceae family, known in Chile as amapola or adormidera (poppy). It was introduced from Europe into Chile, where it grows between the Coquimbo and Los Lagos regions at altitudes up to 500 masl. It is an annual plant that grows up to approximately over 1 m. The leaves are glabrous, and covered with wax which gives them a shiny appearance. The flowers may be white, but are mostly lilac, with a dark violet center, making it an attractive ornamental plant. One of its unique characteristics is the seed capsule, which is globular in shape, with an overlapping disc, and contains numerous dark, tiny seeds—widely used in baking and pastries.

Poppy has been one of the most important medicinal plants in the world for centuries. Poppy is the only plant capable of synthesizing two important narcotic analgesics, namely, morphine and codeine, as well as a variety of other biologically active alkaloids [147]. Figure 4 illustrates the structural relationship among these components. Morphine is a compound first isolated in 1803 by the German pharmacist Friedrich Sertürner [54,148]. Other benzylisoquinoline alkaloids of pharmaceutical importance found in poppy include the antimicrobial agent sanguinarine and the muscle relaxant papaverine and the cough suppressant—and potential anti-carcinogenic—noscapine [149,150,151]. Opioids, in general, have similar side effects, including emesis, cough inhibition, constipation, and respiratory depression [152].

Some recorded cases of death involved poppy extracts or metabolites. In one of these cases, the used agent was morphine [153]. In another case, the victim ingested an infusion of poppy seed combined with benzodiazepine. The cause of death was attributed to the presence of morphine, codeine, and thebaine (contained in the plant infusion) combined with the drug [154]. Another form of consumption is called “rachacha”, a paste prepared by decocting poppy heads until the water has boiled off [155]. There are also reports of direct consumption of poppy seed capsules [156], or consumption of the plant latex, combined with cannabis and/or alcohol [157].

The main symptoms reported in previous cases were seizures and respiratory insufficiency, mimicking an epileptic attack [158]. Toxicological analysis shows the presence of morphine and codeine in blood, urine, and bile. Different concentrations of thebaine, morphine (free), and codeine have been found in samples of peripheral blood, urine, and stomach contents. Other toxicological findings were the presence of metabolites of cocaine and cannabis when poppy was mixed with these drugs [159].

## 4. Interaction with Other Substances

Alcohol (ethanol) and benzodiazepines (in that order) are the psychoactive substances most frequently detected in all cases of death by poisoning [160]. Alcohol is frequently associated with psychotropics in cases of intoxication, especially in juvenile social activities. In cases of suicide, significant levels of alcohol are frequently found in the blood of the deceased. Additionally, some kind of drug abuse is used to take his/her own life [161]. A study carried out in the Los Ríos region of Chile between 1996 and 2008 analyzed 498 deaths by suicide. It was found that approximately 50% of suicides had been carried out under the effects of alcohol. When the data were analyzed by gender, it was shown that 50% of men commit suicide under the influence of alcohol, while women represent only 30% [162]. This is consistent with the recognition that alcohol plays a major role in all patterns of death by poisoning, followed by benzodiazepine [160].

Cases have been reported about extracts of *D. stramonium* being added to alcoholic drinks to increase their effect, which generally ends in intoxication [48]. Gusinde, in 1936, cited by Plowman [74], reported that this combination was also used by inmates, who drank a beverage of *D. stramonium* boiled in wine before being tortured in prisons: “they did not feel any pain, however tight the ropes were pulled”. Something similar was practiced by certain tribes of tropical West Africa, where it was traditional to add *D. stramonium* seeds to the beer or local palm wine to provide a narcotic effect [54].

## 5. Difficulties for Forensic Science or Medical Diagnosis

The use of plant-based drugs implies a series of difficulties including the determination of toxic levels of bioactive ingredients, how a particular plant may have contributed to the intoxication process, and whether a plant was, in fact, the cause of death. The absence of diagnostic pathological findings in autopsies would represent a problem as well [10]. An essential factor in forensic toxicology is the identification and analysis of data. This would allow the investigator to deduce the role played by one or more substances in a case of drug poisoning. However, the interpretation of analytical results is one of the most complex aspects of forensic toxicology. Drug concentration in post-mortem samples does not necessarily reflect the real blood concentration at the moment of death. This is due to variations depending on the sample type and the time interval between death and sampling [163]. Frequently, poisoning is caused not by alkaloids found in the plant but by chemicals absorbed from the environment. Many plants, especially those used in folk medicine, possess a great ability to absorb and accumulate heavy metals in their tissues, such as nickel, copper, lead, cadmium, mercury, and arsenic [164]. These cases have been reported in the United States. For example, a case involved a 5-year-old child whose parents frequently gave him a vitamin mixture composed of Tibetan herbs. After more than four years of constant ingestion, the child manifested symptoms of lead poisoning; finally, it was found he had ingested 63 g of lead. In the US, another case of a 5-year-old child who suffered from bilateral retinoblastoma was also reported. His parents decided to apply a traditional medical treatment from India, which led to arsenic poisoning [10]. In the aforementioned cases, multiple symptomologies, or a variety of symptoms that were either unclear or hardly attributable to a concrete fact, were present. This situation delays diagnosis and, therefore, increases the risk of a fatal outcome. Diagnosis is made much easier when there is an eyewitness who saw the patient ingesting some plant or beverage.

## 6. Toxicovigilance and Forensic Botany

Globally, toxicovigilance has advanced through the incorporation of big data analytics and artificial intelligence, which improves the identification of toxicological risks and the assessment of harmful agents in humans [165]. Poison control centers are essential for collecting and analyzing exposure data to support prevention and management strategies.

In South America, toxicovigilance has progressed considerably, though its implementation remains heterogeneous across countries. The Latin American and Caribbean Toxicology Network (RETOXLAC), supported by the Pan American Health Organization (PAHO), since 1999, has played a central role in regional coordination. RETOXLAC promotes information exchange, standardization of intoxication reporting, and capacity-building in Toxicological Information and Advisory Centers (CIATs) and Clinical Toxicology Analysis Laboratories (LACTs) [166].

In Chile, the Institute of Public Health (ISP) has produced detailed monographs on 47 toxic plant species, providing data on their active compounds and adverse effects [29]. In Argentina, the Toxicology Service of the Rosario Children’s Sanatorium (Sertox) reported that plant poisonings accounted for 0.62% of consultations between 1990 and 2002, underscoring the importance of accurate botanical identification [167]. Brazil’s National System for Toxic-Pharmacological Information (SINITOX) [168], in place since 1980, coordinates a network of regional centers that collect toxicological data and support national surveillance [169]. Uruguay maintains an active CIAT that contributes to RETOXLAC and leads studies on pesticide and chemical intoxication [170]. Paraguay has implemented a poisoning notification system in primary care centers in collaboration with organizations such as ALTERVIDA, although it still faces challenges in training and institutional prioritization [171]. In 2024, the Dominican Republic, with support from the Pan American Health Organization (PAHO), launched a new Toxicological Information Center to improve the management and prevention of poisoning incidents [172].

In parallel, forensic botany has experienced substantial global progress [173], particularly through the adoption of DNA barcoding and environmental DNA (eDNA) techniques for the accurate identification of plant species involved in criminal investigations [174,175]. These methods have proven valuable in solving cases related to environmental crimes, illegal species trade, and homicides.

In Chile, significant efforts have also been made in forensic botany. A project led by the Catholic University of Temuco between 2012 and 2015, in collaboration with the Chilean Investigative Police (PDI), developed and validated technical-scientific protocols to support criminal investigations using plant-based evidence. One resulting study highlighted *Aristotelia. chilensis* as a promising forensic bioindicator, with potential applications in crime scene reconstruction [13].

Together, these initiatives underscore a growing regional commitment to toxicovigilance and forensic botany. However, sustained investment in infrastructure and specialized training remains essential to ensure their effective implementation across South America.

## 7. Conclusions

The analysis of eight toxic and hallucinogenic plant species present in southern Chile reveals a significant gap in forensic reporting and toxicovigilance. While some of these species are well-documented in clinical and scientific literature, local forensic data remain scarce or anecdotal. The presence of highly active compounds—such as tropane alkaloids, coniine, colchicine, taxine, and morphine derivatives—demonstrates the potential for serious health risks, whether due to accidental exposure, intentional misuse, or their involvement in criminal contexts such as drug-facilitated crimes. In several cases, plant access is unregulated, and public awareness of their toxicity remains limited.

Although Chile has made progress in forensic botany and in the implementation of poisoning control systems, there remains an opportunity to further strengthen these areas by adopting advanced technologies and improving case surveillance and reporting related to toxic plants. Continued collaboration among academic institutions, government agencies, and international organizations will be crucial to enhancing the country’s capacity for poisoning prevention and management, as well as for the application of forensic botany in criminal investigations.

This review highlights the urgent need to strengthen forensic scientific documentation systems for plant-related poisonings in Chile, including the creation of clear and accessible databases, the effective dissemination of information among law enforcement and medical centers, and the promotion of specialized training for medical and legal professionals. Public education campaigns on phytotoxicity are also essential. Improving awareness, case detection, and interinstitutional coordination will be crucial to mitigating associated health risks and supporting the effective use of forensic science in toxicological investigations involving plant-derived compounds.

## Figures and Tables

**Table 1:** Search terms used in the literature review.

Category	Search Terms (Examples)
Scientific Names	*Datura stramonium*, *Latua pubiflora*, *Atropa belladonna*, etc.
Common Names	Chamico, Latué, Belladona, etc.
Toxicology Terms	Toxicity, Poisoning, Toxic plants, Poisonous plants of Chile, Chemical submission
Forensic Relevance	Forensic cases, Suicide, Voluntary ingestion, Involuntary ingestion, Criminal poisoning

**Table 2:** Definition and differentiation of key concepts applied to forensic-interest plants.

Term	Definition	Example from this Review	Clarification
Toxic	A substance that may cause physiological or biochemical harm, depending on the dose and exposure.	Any plant containing bioactive alkaloids.	Not all toxic substances are lethal; toxicity depends on dose and context.
Poisonous	A substance that causes severe or fatal effects even at low doses when ingested or absorbed.	*Conium maculatum*, *Taxus baccata*, *Colchicum autumnale*.	A more specific term, usually associated with acute or lethal effects.
Hallucinogenic	A substance that alters perception, consciousness, or cognition via central nervous system action.	*Datura stramonium*, *Brugmansia* spp., *Atropa belladonna*.	Not all hallucinogens are poisonous; risks increase with improper use or overdose.
Sedative	A substance that depresses the central nervous system, causing calmness or sleep.	*Papaver somniferum* (morphine, codeine).	Therapeutic as analgesic and antitussive; becomes toxic at high doses due to respiratory depression, especially when combined with other central nervous system (CNS) depressants.

**Table 3:** Botanical species with toxic active compounds present in southern Chile.

Species	Family	Common Name	Origin	Most Toxic Part of the Plant
*Datura stramonium*	*Solanaceae*	Chamico, Miyaya	Native	Seeds and leaves
*Latua pubiflora*	*Solanaceae*	Latué, palo de brujas [witches’ tree]	Native, endemic	Entire plant, especially leaves and root
*Atropa belladonna*	*Solanaceae*	Belladonna	Introduced	Fruits (berries), roots, and leaves
*Brugmansia vulcanicola*	*Solanaceae*	Floripondio (handkerchief tree)	Native	Leaves and flowers
*Conium maculatum*	*Apiaceae*	Cicuta (hemlock)	Introduced	Entire plant (especially fruits)
*Colchicinum autumnale*	*Liliaceae*	Colchicina	Introduced	Seeds and bulbs (corms)
*Taxus baccata*	*Taxaceae*	Tejo (yew)	Introduced	Seeds (inside the red aril) and leave
*Papaver somniferum*	*Papaveraceae*	Amapola, adormidera (opium poppy)	Introduced	Latex (raw opium), capsules

**Table 4:** Toxicological and pharmacological profile of selected poisonous plant species.

Species	Main Toxic Substances	Pharmacological Property	Pharmacological Activity	Main Symptoms	Severity
*Datura stramonium*	Atropine, scopolamine	Poisonous and hallucinogenic	Anticholinergic, deliriant, CNS depressant	Hallucinations, mydriasis, tachycardia, dry mouth, delirium, seizures	High—Potentially lethal
*Latua pubiflora*	Atropine, scopolamine	Poisonous, hallucinogenic	Anticholinergic, deliriant	Confusion, hallucinations, paralysis, amnesia, coma	High
*Atropa belladonna*	Atropine, scopolamine, belladonnine	Hallucinogenic, poisonous	Anticholinergic, sedative, mydriatic	Dry mouth, blurred vision, delirium, fever, arrhythmias, coma	High
*Brugmansia vulcanicola*	Atropine, scopolamine	Poisonous, hallucinogenic	Anticholinergic, deliriant	Disorientation, hallucinations, hyperthermia, tachycardia, coma	High
*Conium maculatum*	Coniine, γ-coniceine	Poisonous	Nicotinic receptor agonist, neuromuscular blocker	Ascending paralysis, blurred vision, respiratory difficulty, respiratory arrest	Very high—lethal
*Colchicinum autumnale*	Colchicine	Poisonous	Antimitotic, cytotoxic	Nausea, vomiting, severe diarrhea, multiorgan failure, medullary depression	Very high—lethal
*Taxus baccata*	Taxine, 3,5-DMP (3,5-dimethoxy phenol)	Poisonous	Cardiotoxic, CNS depressant	Nausea, hypotension, bradycardia, arrhythmias, cardiac arrest	Very high—lethal
*Papaver somniferum*	Morphine, codeine, thebaine	Sedative	Opioid analgesic, CNS depressant	Somnolencia, depresión respiratoria, coma, miosis, paro respiratorio	High—lethal at high doses

## Data Availability

Data are contained within the article.
